# Conduction and Gating Properties of the TRAAK Channel from Molecular Dynamics Simulations with Different Force Fields

**DOI:** 10.1021/acs.jcim.0c01179

**Published:** 2020-12-09

**Authors:** Riccardo Ocello, Simone Furini, Francesca Lugli, Maurizio Recanatini, Carmen Domene, Matteo Masetti

**Affiliations:** † Department of Pharmacy and Biotechnology, Alma Mater Studiorum−Università di Bologna, via Belmeloro 6, 40126 Bologna, Italy; ‡ Department of Medical Biotechnologies, University of Siena, 53100 Siena, Italy; § Department of Chemistry “G. Ciamician”, Alma Mater Studiorum—Università di Bologna, via Selmi 2, 40126 Bologna, Italy; ∥ Department of Chemistry, University of Bath, Claverton Down, BA2 7AY Bath, U.K.; ⊥ Department of Chemistry, University of Oxford, Mansfield Road, OX1 3TA Oxford, U.K.

## Abstract

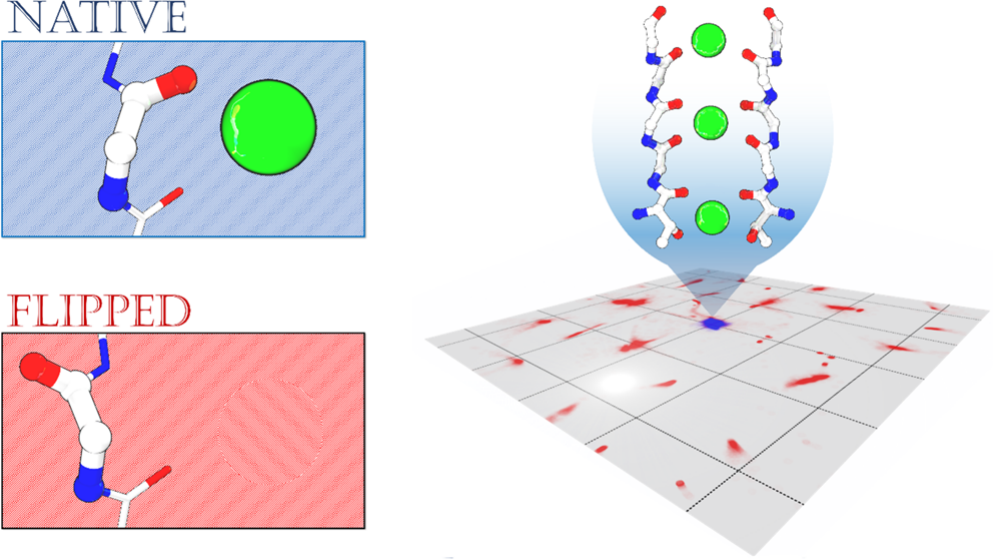

In recent years, the K2P family of potassium channels has been the subject of intense research activity. Owing to the complex function and regulation of this family of ion channels, it is common practice to complement experimental findings with the atomistic description provided by computational approaches such as molecular dynamics (MD) simulations, especially, in light of the unprecedented timescales accessible at present. However, despite recent substantial improvements, the accuracy of MD simulations is still undermined by the intrinsic limitations of force fields. Here, we systematically assessed the performance of the most popular force fields employed to study ion channels at timescales that are orders of magnitude greater than the ones accessible when these energy functions were first developed. Using 32 μs of trajectories, we investigated the dynamics of a member of the K2P ion channel family, the TRAAK channel, using two established force fields in simulations of biological systems: AMBER and CHARMM. We found that while results are comparable on the nanosecond timescales, significant inconsistencies arise at microsecond timescales.

## Introduction

**Figure 1:**
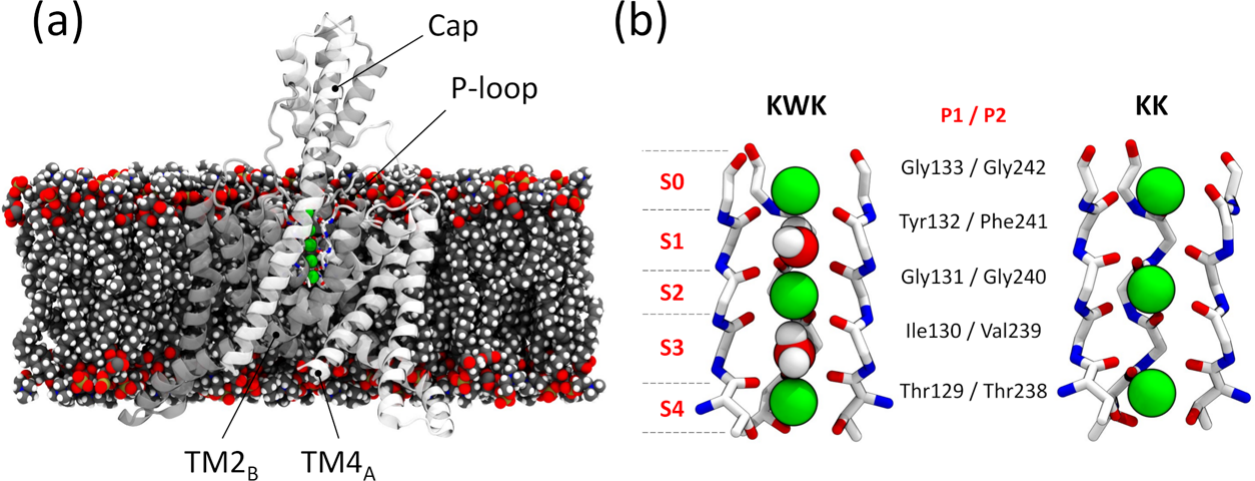
(a) Representative snapshot of the simulation system where the protein is shown in cartoon representation, and the lipid molecules and the potassium ions are represented with van der Waals spheres. The two subunits of the channel are displayed in white and gray colors. Water molecules and bulk ions are omitted for the sake of clarity. (b) Close-up view of the SF in the two initial configurations considered in this study (only three out of the four chains are shown). The residue name of the amino acids that contribute to the SF from the two P-loop regions of each subunit (P1 and P2) are also shown. Ion binding sites are labeled S0 to S4.

Potassium channels are integral membrane proteins responsible for the efficient and selective conduction of K^+^ ions across the membrane. Depending on their overall architecture and functionality, three main classes can be distinguished: voltage-gated (Kv), inward rectifier (Kir), and two-pore-domain (K2P) K^+^-channels.^1^ Even though the Kv and Kir are the most studied families, a growing interest in K2P channels has recently arisen.^2^ Unlike the vast majority of homotetrameric K^+^-channels, members of the K2P family are homodimers but they are nonetheless arranged in a 4-fold-like symmetry around the pore axis of the protein (see Figure 1a).^3^ Another of their peculiar features is the presence of an extracellular domain, known as the “cap”, whose function and specific role in ion conduction, if any, is yet to be established.^4^ At the extracellular entrance of the pore, each subunit carries two P-loop regions (P1 and P2) with distinct signature sequence motifs. Like in canonical K^+^-channels, these are arranged to form a narrow constriction, the selectivity filter (SF), where a series of binding sites for potassium ions are hosted (S0 to S4 starting from the extracellular side, see Figure 1b).^5^ Each site consists of eight coordinating oxygen atoms of the backbone, except for S4, which is made by backbone and sidechain oxygen atoms of threonine residues, and S0, which contributes only with four coordinating oxygens from the protein.^5,6^ K2P channels are responsible for setting the resting potential of the membrane close to the electrochemical equilibrium for K^+^ ions and counterbalancing membrane depolarization.^7^ Their activity is influenced by several physicochemical stimuli, including temperature, pH, membrane stretch, and even changes in the membrane potential, just to mention a few.^7,8^ This multifactorial regulation is peculiar to this family of channels and its complexity has only recently started to be fully acknowledged. From this standpoint, molecular dynamics (MD) simulations and related methods are becoming essential tools to complement experimental observations and to characterize the structural and molecular mechanisms implicated in the functionality of K2P channels.^9−14^

MD simulations have traditionally played a key role in the field of ion channel research as aspects related to conduction and selectivity are governed by detailed atomic-level features that cannot be directly observed by experiments.^15^ Since the pioneering studies on the prototypical KcsA K^+^-channel, MD simulations have been widely used for this purpose,^16−24^ and they have proved instrumental in portraying two alternative mechanisms of ion conduction differing by the presence^17,19,20^ or absence^25,26^ of interposed water molecules (KWK and KK mechanisms, respectively, see Figure 1b). Despite their popularity, MD simulation methods have historically suffered from two main drawbacks. The first is related to the time-consuming nature of the calculations involved which limits the observation of events to the timescales that can be accessed through the available computational resources. Owing to recent software and hardware improvements,^27^ together with advances in methods and protocols suited to enhance sampling efficiency,^28,29^ multi-microsecond-long simulations can be routinely achieved in high-performance computational facilities at present. To date, MD simulations have characterized at a fully atomistic level several functional aspects of K^+^-channels, including inactivation and gating processes.^9,12,30−32^ The second drawback of MD simulations concerns their reliability, which is dictated by both the quality of the underlying potential energy function and the level of realism used to describe the model system under investigation. The latter issue is related to the choice of fixed protonation states for titrable residues (e.g., Asp, Glu, His) as well as oversimplified descriptions of lipid composition for membrane systems. Concerning force fields, while they have reached a remarkable maturity in the field of biomolecular simulations,^33^ they are still severely challenged by the complexity of specific interactions like those taking place in the SF of ion channels. This is particularly relevant when it comes to simulating ion conduction where the detailed balance of attractive and repulsive electrostatic interactions, together with the lack of explicit treatment of polarization effects, push empirical force fields to their limits. Furthermore, state-of-the-art simulations, those reaching microsecond timescales, disclose artifacts or suboptimal features of classical force fields that were previously unnoticed simply by virtue of the shorter timescales attainable.

AMBER and CHARMM are two distinct families of force fields that have gained great popularity in the field of MD simulations of ion channels. While they provide similar results when simulations in the nanosecond timescales are considered, significant differences emerge in the microsecond regime. In a recent study, we have demonstrated that the structure of the SF in the KcsA K^+^-channel is particularly sensitive to the choice of the force field, displaying remarkable stability with AMBER and collapsing in simulations performed with CHARMM.^34^ To understand if such a behavior is specific to KcsA or if, in contrast, it reflects a more general trend, here, we extended the comparison of the two force fields to the simulations of the TRAAK channel.

TRAAK (TWIK-related arachidonic acid activated K^+^-channel, KNCK4, K_2P_4)^35^ is a member of the TREK subfamily of K2P channels that have recently been the subject of intense experimental and computational investigation.^11,12,36,37^ We opted to study the dynamics and ion conduction of this channel since the SF bears significant differences compared to the sequence of canonical K^+^-channels (T_75_V_76_G_77_Y_78_G_79_ in KcsA). In detail, these include: (i) different sequences in P1 and P2, (ii) V_76_ is replaced by isoleucine in P1, and (iii) Y_78_ is replaced by phenylalanine in P2. In addition, TRAAK is a mechanosensitive channel gated by membrane stretch, the positive curvature of the lipid bilayer, and polyunsaturated fatty acids.^38,39^ Gating upon membrane stretch is a characteristic shared by other members of the TREK subfamily of K2P channels. X-ray structures show that the TM4 helices of these channels can bend around the position of a conserved glycine (Gly268 in TRAAK) giving rise to two distinct conformational states, most likely involved in mechanosensing. These states are referred to as “up” and “down” depending on the position adopted by the TM4 helix of one subunit in comparison to the TM2 of the other chain of the channel. In the up state, TM4 and TM2 interact, while in the down state, TM4 is significantly displaced from the other subunit, resulting in a lateral fenestration that opens on the TM region of the channel directly facing the lipid environment. MD simulations have been used to investigate the gating mechanism of this family of channels, and it is therefore important to perform a comparative study of the behavior of the transmembrane segments directly involved in the gating process with the two force fields. Analysis of microsecond-long trajectories of ion conduction carried out with starting configurations matching both the KWK and KK mechanisms, for a total simulation time of 32 μs, showed significant differences between AMBER and CHARMM simulations. Taken as a whole, these results confirm the need for reconsidering the performances of widespread empirical force fields in light of currently achievable timescales.

## Methods

### System Setup and MD Simulations

The model of the TRAAK channel was based on the Protein Data Bank entry 4WFE.^38^ The entire transmembrane domain of the channel was considered from residue Arg2 to residue Arg284. The protonation and tautomeric state of residues at pH 7.0 was assigned with PROPKA.^40^ The channel was inserted into a homogeneous 1-palmitoyl-2-oleoyl-*sn*-glycero-3-phosphocholine (POPC) membrane using the CHARMM-GUI webserver^41,42^ according to the information stored on the Orientations of Proteins in Membranes (OPM) database.^43^ The system was solvated using TIP3P water molecules,^44^ and a 200 mM concentration of KCl was added to the simulation box. Potassium ions were manually placed at the SF sites S0, S2, and S4. For simulations starting from the KWK configuration, water molecules were also added at sites S1 and S3. Two force field models belonging to the CHARMM and AMBER families were used: CHARMM36m^45^ and ff14SB,^46^ respectively. Thus, based on the initial configuration of the SF, two subsets of MD simulations were conceived for each force field, hereafter referred to as AMBER-KK, AMBER-KWK, CHARMM-KK, and CHARMM-KWK.

For the CHARMM set of simulations, the standard Lennard-Jones parameters included in the CHARMM36m distribution were used^45^ and van der Waals interactions were switched off between 10 and 12 Å. For the AMBER set of simulations, the Joung and Cheatham parameters for ions were used,^47^ and van der Waals interactions were truncated at 9 Å together with the standard AMBER scaling for 1–4 interactions. Note that the different setups reflect the most commonly used combinations of force fields and parameters adopted by the community, that is the CHARMM force field parameters for protein, lipids, and ions and AMBER for proteins and lipids together with Joung and Cheatham parameters for ions. This choice leads to the use of a different cutoff for nonbonded interactions in both sets of simulations in consistence with those employed in the development of the corresponding force field. The same setups have recently been used for comparing the AMBER and CHARMM force fields in the study of the KcsA channel.^34^ In all the simulations, long-ranged electrostatic interactions were treated with the particle mesh Ewald method using a grid spacing of 1.0 Å,^48^ and the SETTLE algorithm was used to restrain bonds involving hydrogen atoms to use a 2 fs timestep.^49^ The standard CHARMM-GUI protocol whereby the system is heated up and any initial restraints are smoothly released was adopted. The temperature was controlled using a Langevin thermostat with a damping coefficient of 1 ps^–1^, while the pressure was maintained at the constant value of 1 atm by coupling the systems to a Nosé-Hoover Langevin piston, with a damping timescale of 25 ps and a period of 50 ps.^50^ The membrane potential was simulated by applying a constant electric field acting along the whole length (*L*_*z*_) of the *z*-axis of the simulation box. In particular, an electric field, *E*_*z*_, corresponding to two different transmembrane potentials, Δ*V*, of +100 and +200 mV, was applied to all of the atoms of the system^51,52^
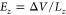
1Production runs were performed in the NVT ensemble at the target temperature of 310 K as previously described.^52,53^ The NAMD-2.12 MD engine was used for all of the simulations with a timestep of 2 fs.^54^ For each set of simulations, two copies were performed at equilibrium conditions and three copies were run under +100 and +200 mV. Every run lasted 1 μs of sampling, amounting to a total simulation time of 32 μs. Data were aggregated unless otherwise stated. A summary of the simulations considered in this study is presented in Table 1.

**Table 1:** Summary of the Simulations Considered in This Study and the Relevant Features of the Different Simulation Runs

force field	initial SF configuration of ions	membrane potential (mV)	replicas	prevalent conformational state^a^	lipids in cavity^b^	conduction events (#)
AMBER	KK	0	#1	up/up	no	0
AMBER	KK	0	#2	up/up	no	0
conductance: –						
AMBER	KK	100	#1	up/up	no	2
AMBER	KK	100	#2	up/up	no	4
AMBER	KK	100	#3	up/up	no	2
conductance: 4.3 ± 1.9 pS						
AMBER	KK	200	#1	up/up	no	3
AMBER	KK	200	#2	up/up	no	11
AMBER	KK	200	#3	up/up	no	8
conductance: 5.9 ± 3.2 pS						
AMBER	KWK	0	#1	up/up	no	0
AMBER	KWK	0	#2	up/up	no	0
conductance: –						
AMBER	KWK	100	#1	up/up	no	1^c^
AMBER	KWK	100	#2	up/up	no	0
AMBER	KWK	100	#3	up/up	no	2^c^
conductance: 1.6 ± 1.6 pS						
AMBER	KWK	200	#1	up/up	no	0
AMBER	KWK	200	#2	up/up	no	0
AMBER	KWK	200	#3	up/down	yes	0
conductance: –						
CHARMM	KK	0	#1	up/down	yes	0
CHARMM	KK	0	#2	up/down	yes	0
conductance: –						
CHARMM	KK	100	#1	up/up	no	0
CHARMM	KK	100	#2	down/up	yes	0
CHARMM	KK	100	#3	up/up	no	0
conductance: –						
CHARMM	KK	200	#1	up/up	no	0
CHARMM	KK	200	#2	down/up	yes	11
CHARMM	KK	200	#3	up/up	no	0
conductance: 2.9 ± 5.1 pS						
CHARMM	KWK	0	#1	up/down	yes	0
CHARMM	KWK	0	#2	up/down	yes	0
conductance: –						
CHARMM	KWK	100	#1	up/up	no	0
CHARMM	KWK	100	#2	up/up	no	0
CHARMM	KWK	100	#3	up/up	no	0
conductance: –						
CHARMM	KWK	200	#1	down/up	yes	0
CHARMM	KWK	200	#2	down/up	yes	0
CHARMM	KWK	200	#3	up/down	yes	0
conductance: –						
Total Simulation Time: 32 μs						

The duration of each simulation was 1 μs amounting to 32 μs. Experimental conductance: 65.4 ± 3.2 pS at +100 mV under symmetrical 150 mM KCl.^70^

a The distance threshold between up and down states is considered to be 8 Å. A conformational state is regarded as prevalent in a single run if it persists by more than 50% of the total simulation time.

b A lipid is considered to be inside the cavity if the headgroup is found at a distance lower than 10 Å in the channel and for more than 50% of the total simulation time.

c The conduction events were sampled after the exit of water molecules from the selectivity filter.

### Analysis of Trajectories

Analysis of the SF stability, backbone dihedral angles, and permeation events was performed with the python libraries available from the MDAnalysis toolkit.^55,56^ The distance of lipid atoms from the channel axis was computed with the driver functionality of PLUMED-2.4.^28^ The ggplot2^57^ package running under the R 4.0.2 programming environment^58^ was used to carry out statistical analysis on the distribution of atomic distances and to plot results.

Two dimensionality reduction methods were used to visualize and compare the conformation of the SF in the different set of simulations: principal component analysis (PCA) and sketch-map.^59,60^ PCA was performed using the dimRed package running under the R 4.0.2 programming environment.^57^ Specifically, the values of the backbone dihedral angles (φ,ψ) of all of the residues of the SF and the χ_1_ angles of Thr129 and Thr238 were converted into sin- and cos-transformed variables prior to carrying out PCA.^61,62^ Conversely, the dihedral angle values of the same set of residues were directly used for the sketch-map analysis. Sketch-map is a nonlinear multidimensional scaling (MDS) method that seeks to preserve middle-ranged distances of the input data structure by minimizing the following stress function^59^
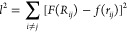
2where *R*_*ij*_ and *r*_*ij*_ are the distances between two points evaluated in the high- and low-dimensional (low-d) space, respectively, while *F* and *f* are two sigmoid functions of the form
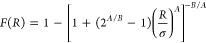
3and
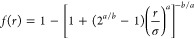
4The effect of the sigmoid functions is to shrink and expand the space for all of the distances below and above the characteristic length determined by σ, respectively. The exponents *A*, *B* and *a*, *b* control the rate at which the distances are switched from 0 to 1 in the high- and low-dimensional (low-d) space, respectively.^59^ To reduce the computational overhead, the sketch-map allows building the low-d embedding using a relevant subset of points from the entire data set (landmark points). Then, the remaining points are projected on the low-d space through a robust out-of-sample procedure.^59,60^ In this work, we used a total number of 1000 landmark points, and the sigmoid functions were tuned with the following parameters: σ = 2.5, *A* = *B* = 12, *a* = 1, and *b* = 2. Trial sketch-map analyses were performed with different combinations of parameters. As expected, the choice of σ was found to be the most critical for achieving an effective dimensionality reduction. The value of 2.5 for σ turned out to be the best compromise to project distinct conformations of the filter in farther regions of the low-d space while preserving a good resolution detail on similar conformations. A density-based cluster analysis (DBSCAN) was then performed on the projections of the trajectories on the low-d space using the dbscan package running under the R 4.0.2 programming environment.^57^ For every subset of simulations, the minimum number of points was set to 3 while the epsilon parameter was equal to 0.8 except for the AMBER-KK series where a value of 0.1 was used. The different parameters used in the latter subset of simulations was necessary to cope with the different data structure showing a higher density of points. The cluster analysis was limited to the most informative region of the sketch-map space, which is the one comprised between −60 and 60 units in both dimensions.

Potential sites for the interaction of nonannular lipids and other hydrophobic molecules with the TRAAK channel have been described in structural biology studies.^38,39^ The movement of lipids toward the cavity of the channel was also analyzed, and the projection of the distance in the membrane plane of selected atoms of a single lipid molecule on the pore axis was considered. In particular, these distances were computed using the XYDISTANCES multicollective variable function available in PLUMED-2.4.^28^ The opening of the fenestration was monitored by evaluating the distance between the TM2 and TM4 helices of the two subunits (A and B) of the channel (TM2_A_-TM4_B_ and TM2_B_-TM4_A_ distances). Specifically, the separation between the helices was determined by computing the distance between the Cα-carbon of Pro155 (TM2) and Ile279 (TM4) as a reference.

## Results

The TRAAK channel in a fully conductive state (up/up configuration of the TM4 helices) was embedded in a lipid membrane and simulated under conditions of symmetrical ion concentration and a positive electrostatic gradient. Two initial configurations of ions and water in the SF were considered according to the KWK and KK models of ion conduction (Figure 1b).^17,19,25,26^ For each of these initial states, three replicas were considered both with the CHARMM36m^45^ and the AMBER ff14SB^46^ force fields (see also Table 1). Furthermore, for each setup, two additional replicas were simulated in equilibrium conditions (Δ*V* = 0 mV) as a control. In the following, we will outline the major similarities and differences from the analysis of the conformations of the selectivity filter, ion conduction, and the behavior of the transmembrane helices of TRAAK that are directly involved in channel gating in the trajectories obtained with both force fields considered.

### Conformational Preference of the SF

In Table 1, a list of the MD simulations performed in this study together with the initial simulation conditions and the number of conduction events recorded on a microsecond timescale for each set of simulations are reported. As expected, no conduction events were recorded under equilibrium conditions (Δ*V* = 0 mV). However, it is remarkable how ion conduction could only be observed in some of the subsets of the simulations performed under electrostatic gradients, namely, AMBER-KK (+100 and +200 mV), AMBER-KWK (+100 mV, but only after the exit of water molecules from sites S2 and S3), and CHARMM-KK (+200 mV). In the case of the CHARMM-KWK subset, ion conduction was never recorded even in the presence of an applied electric field. Moreover, except for AMBER-KK at +200 mV, the number of conduction events was very small. These results not only suggest a dependence of ion conduction on the initial presence of water molecules inside the SF but also a force field-specific behavior that should be carefully analyzed in-depth.

**Figure 2:**
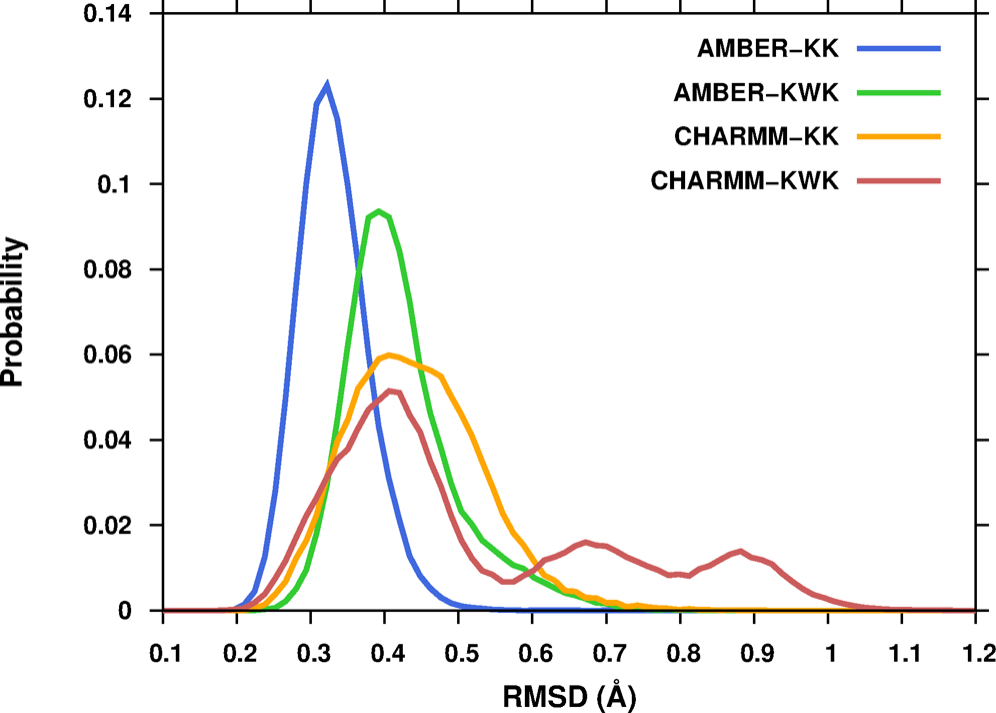
Comparison of the root-mean-square deviation (RMSD) of the SF backbone atoms evaluated in the four sets of simulations. Blue, green, orange, and red correspond to the AMBER-KK, AMBER-KWK, CHARMM-KK, and CHARMM-KWK sets, respectively.

In Figure 2, the root-mean-square deviation (RMSD) of the backbone atoms of the SF is reported for the four subsets of simulations. The data shows that the initial structure of the SF is better preserved when the simulations are performed with the AMBER force field and are initiated from the KK rather than the KWK configuration. It is also found that the RMSD maximum is more sharply peaked in the simulations with the AMBER force field compared to the simulations with CHARMM. The most striking behavior is observed for CHARMM-KWK where three maxima are found, implying a substantial flexibility of the SF that is not detected in the other subsets of simulations.

**Figure 3:**
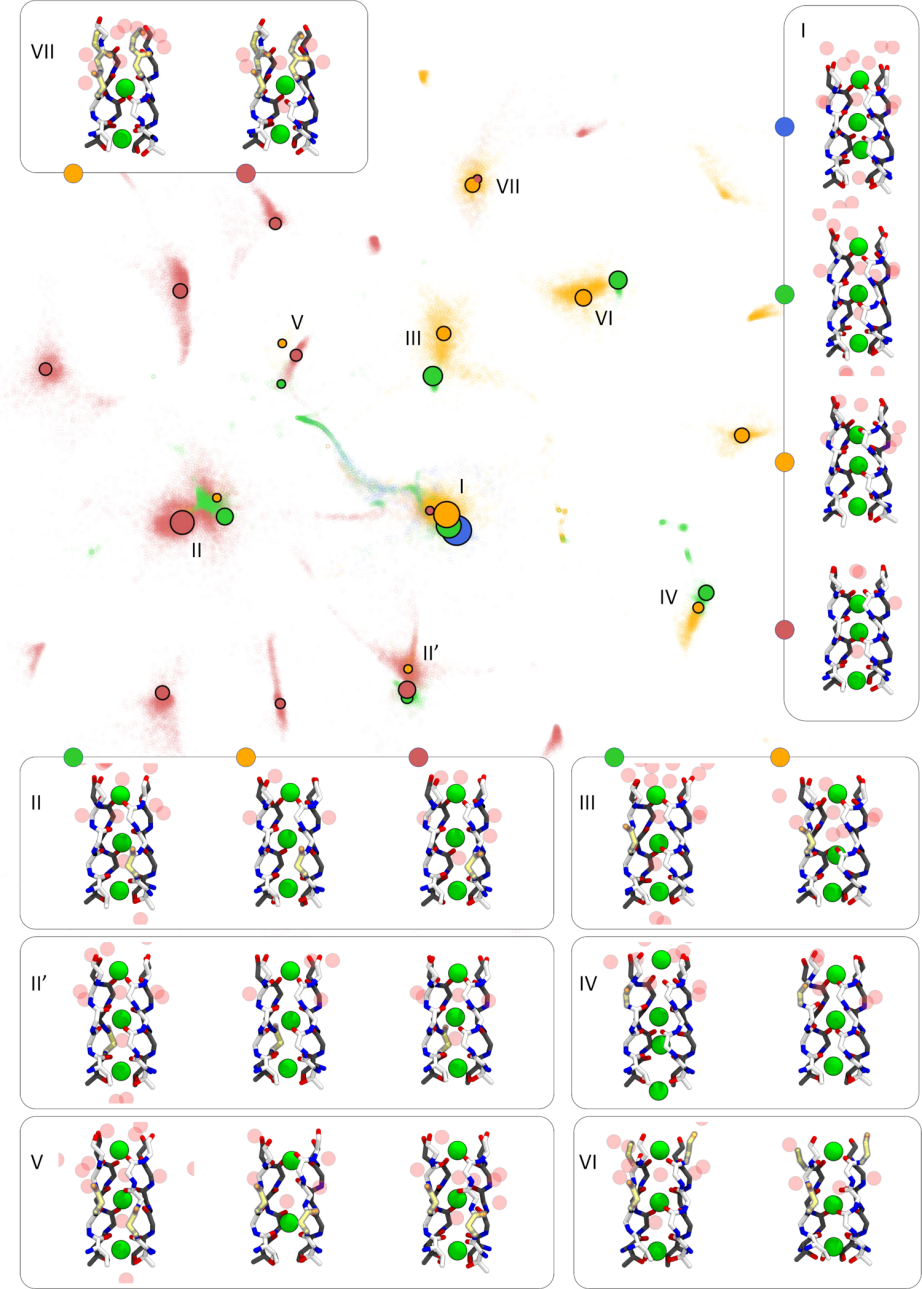
Sketch-map representation of the conformational space sampled by the SF during all of the simulations considered in this study. The recurrent conformational states observed are labeled from I (native/conductive state) to VII. Representative configurations of each conformational state are shown and mapped onto the low-d space with solid colored circles. Blue, green, orange, and red correspond to the AMBER-KK, AMBER-KWK, CHARMM-KK, and CHARMM-KWK sets, respectively. The size of the points is proportional to the populations of the main states using a logarithmic scale. Chains A and B are colored in white and gray, respectively. Flipped carbonyls are highlighted in yellow.

To obtain a more informative picture of the conformational space spanned by the SF in the different subsets of simulations, we resorted to dimensionality reduction techniques such as the well-established PCA and the more recent sketch-map approach.^59^ In all of the cases, dimensionality reduction was performed on the dihedral angles of the SF backbone plus the χ_1_ torsions of Thr129/238 for a total of 44 degrees of freedom as a whole (see the Methods section for further details). In this way, the 44-D conformational space of the SF was reduced to a 2-D map that could be easily displayed and further analyzed aiming at identifying major conformational states and recurrent conformations among the distinct subsets. In Figure S1, the low-d space obtained through PCA is shown. Both with the PCA and sketch-map, the conformations explored by the SF in the AMBER-KK set were restricted to a small portion of the map (blue points) reflecting smaller fluctuations compared to those experienced by the other subsets. The opposite behavior was observed in the CHARMM-KWK set in which the projections of the MD trajectories spanned a substantial area of the low-d space. This behavior can be better appreciated in the sketch-map plot shown in Figure 3. A sketch-map is a nonlinear MDS method that attempts to preserve the middle-ranged distances of the high-dimensional data structure, under the assumption that these encode the most informative features to describe the process under investigation.^59,60^ Thus, in the low-d embedding, points closer than a certain threshold distance (σ = 2.5 in this work) are collapsed, while the remaining ones are separated further away providing an intuitive picture of the whole conformational space sampled by the system. As a result, similar conformational states are grouped together leading to the typical island-like appearance of a sketch-map plot like the one reported in Figure 3. More precisely, regions that are denser in points correspond to distinct metastable states of the SF that were either specific or shared among different subsets of simulations. To better compare the states, a density-based cluster analysis was performed on the projection of the trajectories of each subset of simulations onto the low-d space obtained by the sketch-map. Representative configurations of recurrent conformational states of the SF identified by cluster analysis are shown in the insets of Figure 3. More details regarding the results of cluster analysis can be found in Figure S2 and Table S1 of the Supporting Information, SI.

The most striking feature of the sketch-map plot reported in Figure 3 is that the native and conductive conformation of the SF (state I) is located at the center of the low-d space, while all of the other conformational states around it correspond to conformations where one or more carbonyl groups are flipped away from the channel axis. As reported in Table 2, the canonical conductive state of the SF prevails over other conformations in the AMBER-KK set. In AMBER-KK simulations, only 2.2% of the configurations deviated from the canonical conductive state, by means of carbonyl flipping of glycine residues in S0. States II and II′ represent two symmetric conformations of the SF characterized by the flipping of Gly240 in chains A and B of the channel, respectively, affecting the boundary between S4 and S3 sites. These states were observed in the AMBER-KWK, CHARMM-KK, and CHARMM-KWK sets but with different probabilities (see Table 2). In particular, the total probability of visiting states II-II′ is as low as 1.0% in the case of the CHARMM-KK set and as high as 47.1% in the CHARMM-KWK simulations, yielding the conformation of the SF with one carbonyl flipping at the S4–S3 boundary as the predominant one in the latter subset of simulations. Other conformational states of the SF involving a single carbonyl flipping that can be observed in the low-d space are states III and IV. These are significantly populated only in the AMBER-KWK and CHARMM-KK sets but with similar probability ratios (Table 2) and they involve the carbonyl flipping at the S3–S2 and S1–S0 boundaries, respectively. All of the remaining states identified by the sketch-map represent multiflipped conformations of the SF. For example, in state V, the SF is flipped at both the S4–S3 and S3–S2 boundaries, and while this conformation is observed in all subsets of simulations except the AMBER-KK set, only in the CHARMM-KWK set it significantly contributes to the pool of sampled conformations (4.8%, see Table 2). Conversely, state VI involves one carbonyl flipping at S2–S1 and two symmetric flipping at the S1–S0 boundaries. State VI is observed with similar probabilities only in the AMBER-KWK and CHARMM-KK sets of simulations. Finally, state VII is an example of a multiflipped conformation of the SF that involves all of the boundaries between sites that can be subjected to carbonyl flipping, which are S3–S2, S2–S1, and S1–S0, and it is significantly populated only in the CHARMM-KK set. The states described above represent recurrent conformations that account for a substantial portion of the conformational space of the SF in all of the subsets of simulations except for the CHARMM-KWK subset, in which almost half of the conformations are specific to this simulation condition (44.7%, Table 2). This situation is manifested in the sketch-map plot of Figure 3 in which several spots are only populated by conformations that come from CHARMM-KWK trajectories (see the red dots in Figure 3).

**Table 2:** Recurrent Conformational States of the SF Mapped onto the Sketch-Map Space^a^

		relative population (%)			
		AMBER		CHARMM	
conformational state	carbonyl flipping boundaries	KK	KWK	KK	KWK
I	NA	97.8	43.2	52.3	2.7
II	S4–S3		11.0	0.7	35.5
II′	S4–S3		4.6	0.3	11.6
III	S3–S2		16.6	7.1	
IV	S2–S1		8.5	4.0	
V	S4–S3 and S3–S2		0.4	0.2	4.8
VI	S3–S2 and S1–S0		13.3	10.3	
VII	S0–S1, S1–S2, S2–S3			7.6	0.7
others	NA	2.2	2.4	17.5	44.7

a Full results of cluster analysis are reported in Table S1.

### Stability of the TM Region of the Channel and Interactions with Lipid Molecules

**Figure 4:**
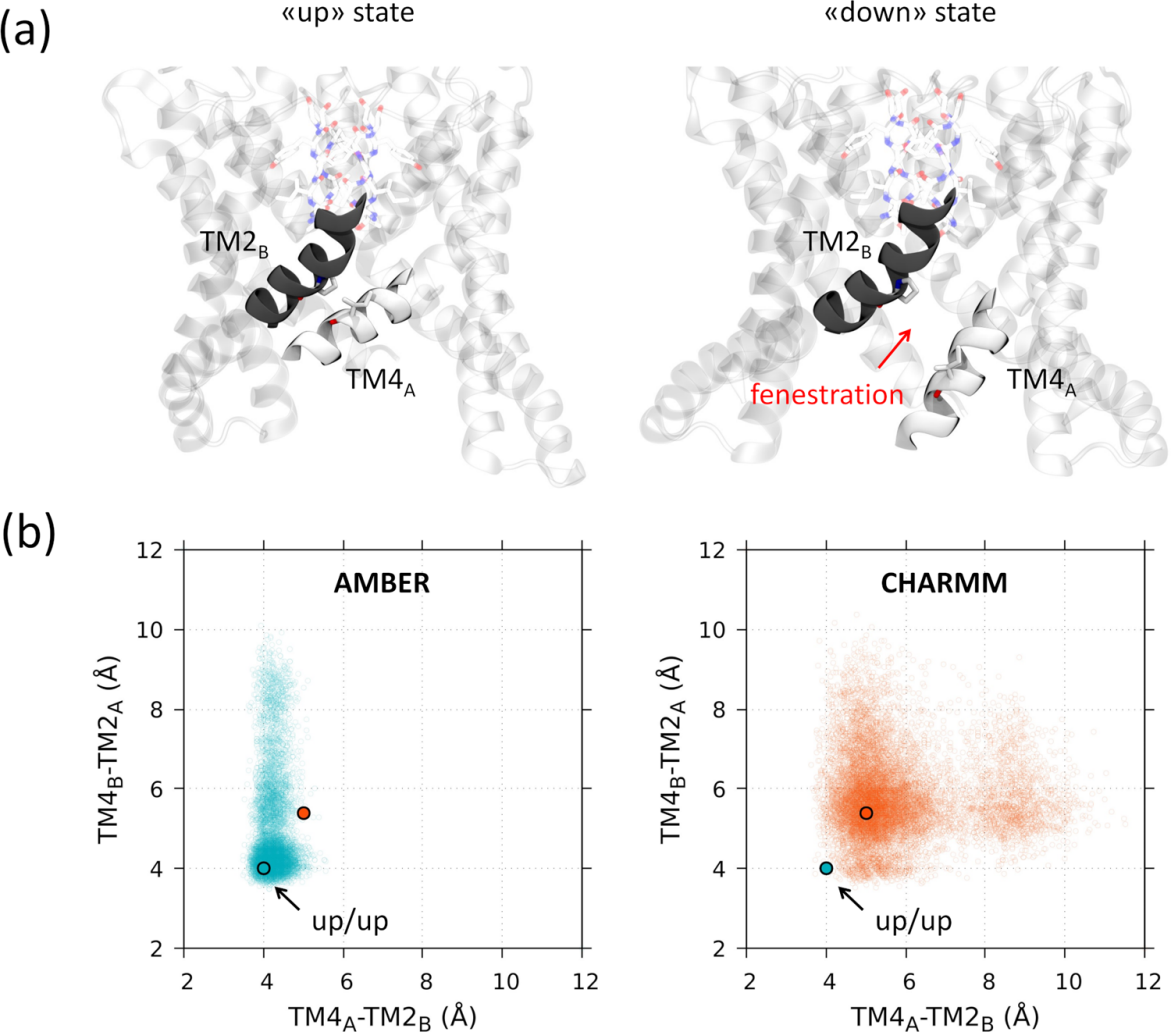
(a) Representative configuration of the up and down states of the protein at one of the interfaces between the two subunits of the channel. The fenestration that appears in the down state at the TM2-TM4 interface. (b) Scatter plots of the distance between the TM2 and TM4 helices of different subunits obtained from the AMBER and CHARMM sets of simulations are highlighted. Blue and orange circles correspond to the maxima of the AMBER and CHARMM distributions, respectively. A and B refer to the two subunits of the channel.

Like the other members of the TREK subfamily of K2P channels, TRAAK is gated by membrane stretch and the positive curvature of the membrane. Even though some contradictory findings have been reported,^38,63^ it is widely accepted that the active state of the channel is represented by the up/up conformation, with the TM4 helices of both subunits kinked upward (Figure 4a), while the down state is regarded as a low conductance one. According to a proposed gating hypothesis, the low conductance associated with the down state can be explained by impairment of ion conduction due to the presence of lipid tails reaching the cavity through the lateral fenestrations.^37^ Upon membrane stretch, the hindrance imposed by the lipids would be released, and the up/up state with sealed fenestrations would be adopted. More recently, an allosteric effect directly linking the “down to up” transition of TM4 with the activation of the SF has been proposed^64−66^ and investigated by several groups using different computational approaches.^67−69^ In this model, the SF would enter into a nonconductive state when the down conformation of TM4 is adopted, resembling the C-type inactivation process observed in Kv channels.^66^

In Figure 4b, a scatter plot is reported showing the distance between the TM4 and TM2 helices calculated for both subunits (i.e., TM4_A_-TM2_B_ and TM4_B_-TM2_A_) in the sets of simulations performed with the AMBER and CHARMM force fields (see also the individual plots for each simulation run in Figure S3). When the two helices interact, the distance between the two reference carbon atoms (see Methods for further details) approaches a value close to 4 Å (up state), while distances above 8 Å are an indication of a substantial separation between the helices with the concurrent opening of the fenestration (down state). All of the simulations were initiated from a fully conductive up/up state, and in both sets of simulations, “up to down” transitions were recorded (one and five transitions in the AMBER and CHARMM simulations, respectively, see Table 1). Notably no backward transitions to the up/up state were ever sampled in this study. As the plot in Figure 4b shows, in the AMBER set of simulations, there is a more pronounced tendency to preserve the up/up state compared to the CHARMM set. Regardless of the number of complete up to down transition events, which are more frequently sampled with the CHARMM force field, the maximum in the distribution of the distances is found at about 4 Å in the AMBER simulations. This value increases by more than 1 Å in the CHARMM simulations which illustrated that the initial conditions are better preserved with the AMBER force field.

**Figure 5:**
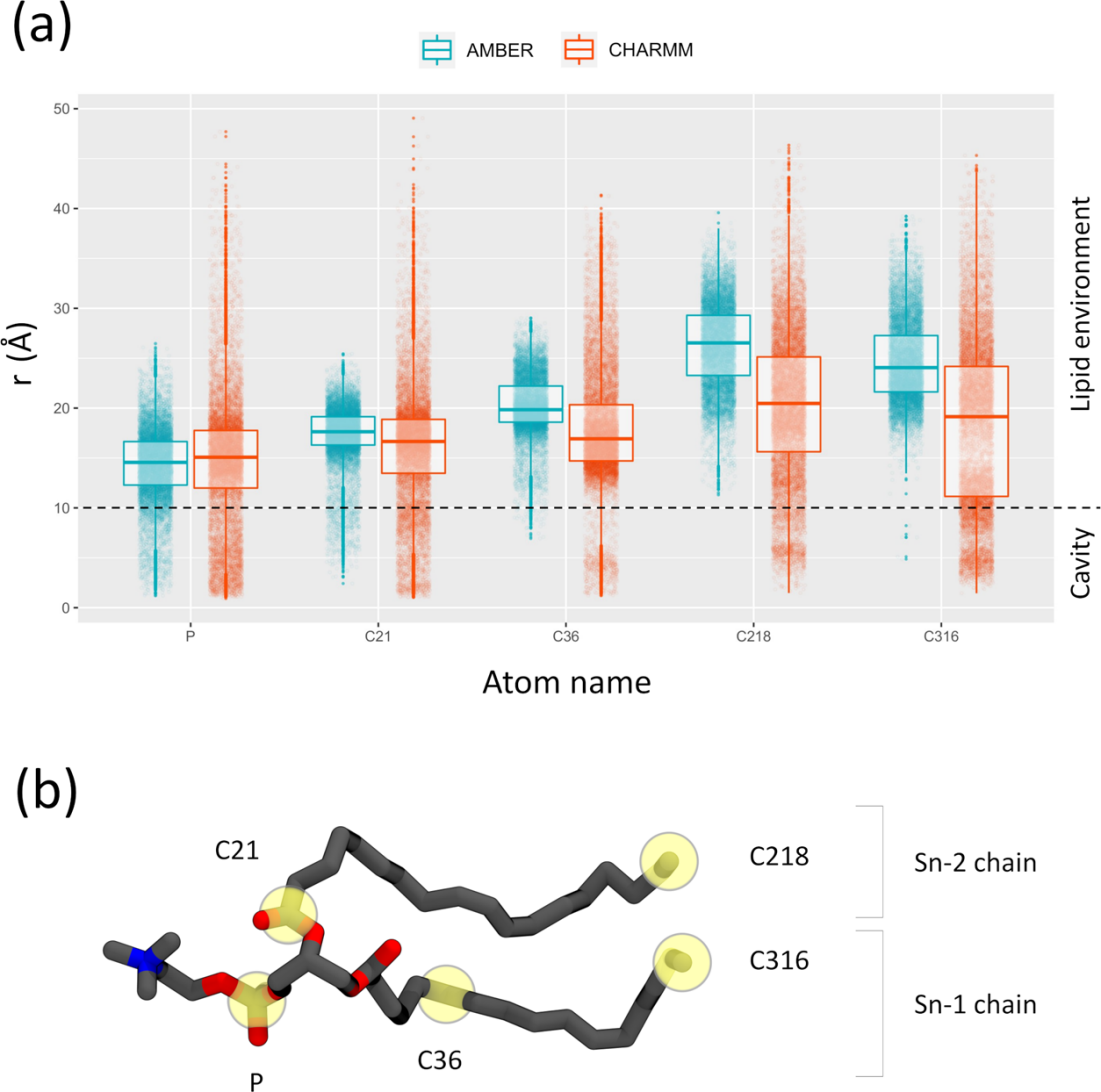
(a) Presence of lipid atoms in the cavity of the TRAAK channel in the AMBER and CHARMM sets of simulations. (b) POPC molecule in stick representation highlighting in yellow the atoms used in the analysis presented in (a) with their corresponding labels.

**Figure 6:**
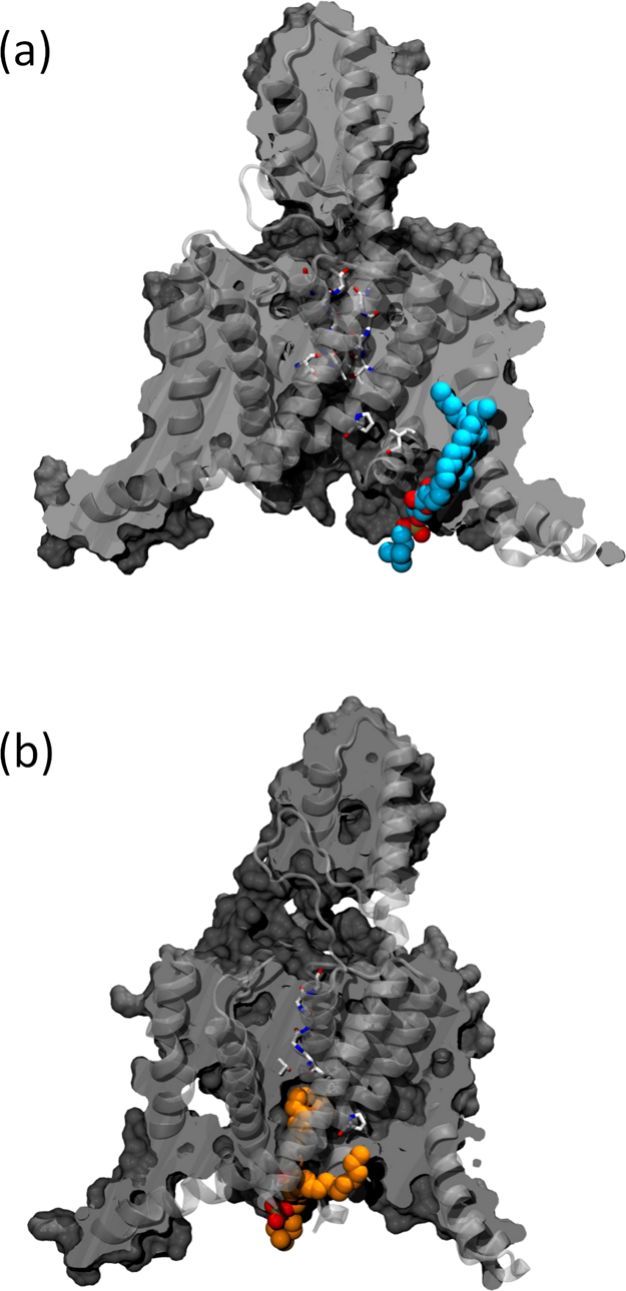
Comparison between typical configurations of lipids facing the internal cavity of the channel obtained with the (a) AMBER and (b) CHARMM force fields. The orientation of the protein is identical in both snapshots. Lipids are depicted in van der Waals representation in blue and orange, respectively. The protein is depicted in gray and the up state is shown in (a), while the down state is displayed in (b).

The conformational state adopted by TRAAK in MD simulation is influenced by the behavior of lipid molecules at the interfaces between the two subunits of the channel. In Figure 5a, the probability of observing a series of reference atoms of a lipid molecule at a given distance from the channel axis in the two sets of simulations is shown. A representative lipid molecule with the atoms used for the analysis is shown in Figure 5b. The degree of penetration of lipid molecules inside the pore of the channel was measured by considering the boundary between the cavity of the channel and the membrane environment at about 10 Å. We observed a substantial penetration of lipids in the simulations performed with the CHARMM force field (see the lower tail of the distribution for atoms P, C21, C36, C218, and C316), while this behavior is much less pronounced in the simulations with the AMBER force field. Values of the distance between the P atoms of the lipids and the pore axis lower than 10 Å observed in the AMBER set of simulations are associated with configurations where the POPC headgroup only slightly faces the cavity from the intracellular side of the membrane (see Figure 6a). Conversely, in the CHARMM simulations, lipids enter the cavity in an almost complete way, remaining anchored to the membrane through the Sn-1 chain (see the distribution of atom C316 in Figures 5a and 6b) and occasionally, pointing the headgroup toward the SF. In spite of this, lipid tails are frequently observed in the cavity even without invoking full lipid excursions. However, as previously reported, this occurrence is unlikely to have any functional implication in ion conduction.^36^ The aggregated data shown in Figure 5a is also reported as individual plots for each simulation run in Figures S4 and S5. From these plots, it can be observed that most of the lipid occlusion events sampled in the CHARMM simulations showed the tendency of a single POPC molecule to enter the cavity entirely (see Figures S5 and S6).

## Discussion and Conclusions

The microsecond-long MD simulations performed in this study under different initial conditions facilitate exploring the effects of either vacancies or water molecules inside the SF and the choice of the force field in the simulation of ion conduction in the TRAAK channel. The most important observation emerging from our data is that the combination of the AMBER force field with the absence of water molecules inside the SF as the initial configuration is a requirement to obtain a significant number of conduction events at both +100 and +200 mV. This occurrence is strongly related to the fact that only in this subset of simulations, the native conductive state of the SF was preserved in the vast majority of the MD runs (more than 95% of the total simulation time, see Table 2). Conversely, when simulations performed with the same force field are initiated from the KWK configuration, the probability of finding the filter in the native conductive configuration drops at about 43% of the total simulation time and so does the total number of conduction events (from 8 to 3 and from 22 to 0 at +100 and +200 mV, respectively). Interestingly, the other significantly populated states observed in this set of simulations mostly involve carbonyl flipping at the S4–S3 boundary (states II and II′, 15.6% in total) or the S3–S2 boundary (state III, 16.5%), even though other multiflipped states involving these coordination states can be found (see Table 2). The same flipping is observed in CHARMM-KWK where states II and II′ account for about 47% of all of the conformations visited by the filter, and in CHARMM-KK but with much lower probabilities. This is an indication that the presence of water molecules triggers the conformational change that brings the SF away from the native conductive state. Once flipping at the S4–S3 boundary occurs with a water molecule in S3, it is difficult to recover the conductive state in a microsecond regime. Moreover, we highlight that all of the conduction events observed in this study took place according to the KK mechanism of ion conduction. Notably, the conduction events recorded for AMBER-KWK at +100 mV were observed after the expulsion of the intercalating water molecule from the S3-binding site. The same behavior was not observed in the CHARMM-KWK subset of simulations, where either the corresponding water molecule remained in the SF or the SF became too unstable to allow ion conduction. The observations described above are consistent with the results of a recent study performed on the KcsA channel.^34^

Concerning the comparison between simulations performed under the same initial conditions but employing different force fields, the most striking result is that the probability of finding the SF in the native conformation is reduced by half going from the AMBER to the CHARMM force field, when the KK configuration is chosen to start with, and conduction is virtually suppressed if the starting configuration is the KWK one. This is a clear indication that AMBER and CHARMM force fields behave differently on a microsecond timescale, highlighting an incipient instability of the SF when the CHARMM force field is considered that can be critical for describing the dynamics of the system under investigation. These findings are in line with the results obtained from the comparison of the two force fields in preserving the native state of the SF for the KcsA channel.^34^ However, while in the case of KcsA the CHARMM force field leads to a full collapse of the SF on the microsecond timescales regardless of the choice of Lennard-Jones parameters for the ions, in the present work the collapsed state of the SF was actually never sampled. This observation suggests that the choice of the force field might influence the structural properties of the simulated channels in a subtler way than it was observed in the other study, also showing a case-dependent behavior. Even though the main goal of this work was to highlight the different states of the SF sampled under different initial conditions, we also attempted to estimate the conductance of the channel using the number of ion translocation events recorded in the independent MD runs (Table 1). The estimated conductance in the AMBER-KK subset of simulations was found to be 4.3 ± 1.9 pS at +100 mV. Thus, the experimental conductance of 65.4 ± 3.2 pS at +100 mV under symmetrical 150 mM KCl is underestimated by more than 1 order of magnitude.^70^ Again, the discrepancies between the experimental and estimated values of the conductance are in line with those obtained for the KcsA channel from simulations performed with the AMBER force field and under the same transmembrane potentials.^34^

Since TRAAK is activated by polymodular stimuli including membrane stretch, in this work, we have also investigated the behavior of the TM helices directly involved in the gating functionality of the channel. Our data clearly suggest that the initial conductive state of the channel (up/up state) is better preserved in microsecond-long simulations employing the AMBER force field. Indeed, while up to down transitions are found in both sets of simulations, these are more frequently observed in the CHARMM simulations. In addition, even when configurations consistent with the up/up state are considered, these appear to be intrinsically less structured in the CHARMM trajectories compared to AMBER ones. This is evident in the broader distributions of distances between the transmembrane helices TM2 and TM4, shown in Figure 4b. It is noticeable that without imposing any membrane stretch to keep the channel in an active conformation, the up to down conformational transition is expected to occur. However, the rate at which this event is observed and its manifestation in several of the CHARMM trajectories highlight a clear difference of this force field in describing the conformational dynamics of the TM4 helix in comparison to the AMBER force field. Results obtained with the AMBER force field are in line with those previously reported by Harrigan et al. in simulations of the closely related TREK-2 channel using the same AMBER force field.^67^ These authors described recurrent and reversible activation events of the channel taking place on a multi-microsecond timescale, the down conformation being the preferred state under equilibrium pressure conditions.^67^ The channel activates upon membrane stretch, so in equilibrium conditions (without stretch), the closed state, corresponding to the down conformation, should be the most likely conformational state.

In the present study, the lower structural stability of the TM region of the channel experienced in the simulations using the CHARMM force field seems to be correlated with an increased probability of lipids reaching the cavity from the open fenestrations present between TM2 and TM4 in the down state. Notably, in the case of CHARMM, these events involve the insertion of almost an entire lipid molecule into the cavity, with the headgroup directly facing the bottom of the S4 site of the SF. Since the presence of lipid tails in the cavity of this subfamily of K2P channels has been suggested to be implicated in the mechanosensing gating mechanism, our results suggest that care must be taken when interpreting data sets from MD simulations. On a final note, we would like to emphasize that the above observations are based on the assumption of a causative relationship between the stability of the protein and the presence of lipids inside the cavity of the channel. However, the two sets of simulations (AMBER and CHARMM) also differ in the force field parameters describing the lipid molecules. Therefore, while it is unlikely that lipid penetration and conformational changes of the TM4 helix are not correlated, this hypothesis cannot be ruled out, nor that the diverging behavior of the TM helices of the protein can be an effect of different lipid parametrization.

Currently, MD simulations are widely employed to study biomolecular systems at a fully atomistic level. The ever-increasing availability of high-performance computational facilities is narrowing the gap between the timescales able to be sampled by simulations and experiments. This, in turn, expands the scope of the computational investigations considered and allows us to benchmark the force field developed in the 1980s using picosecond timescales for the study of processes occurring on much longer timescales. In this work, we compared results from the AMBER and CHARMM force fields with the aim to test their ability to describe several functional properties of the TRAAK channel, including the stability of the SF, the occurrence of ion permeation, and the preferential states adopted by the transmembrane regions of the protein directly involved in gating. While these two force fields behave similarly on the nanosecond regime, results presented in this study demonstrate that they render a strikingly dissimilar picture of the channel dynamics on longer timescales. Even though simulations performed with the AMBER force field under applied membrane potentials of magnitudes comparable to experimental ones provide a more realistic description of ion conduction for the TRAAK channel over the CHARMM force field, the computed values of conductance are still underestimated by an order of magnitude, leaving room for further development in the field.

## Supporting Information Available

Relative population of the main clusters identified from the projection of each subset of trajectories onto the sketch-map low-d space (Table S1); principal component analysis carried out on the degrees of freedom of the SF (Figure S1); density-based cluster anlaysis (Figure S2); scatter plots of the TM2-TM4 distances (Figure S3); time evolution of lipids entering the cavity (Figures S4 and S5) (PDF)

## References

[ref1] Buckingham S. D.; Kidd J. F.; Law R. J.; Franks C. J.; Sattelle D. B. Structure and function of two-pore-domain K^+^ channels: contributions from genetic model organisms. Trends Pharmacol. Sci. 2005, 26, 361–367. 10.1016/j.tips.2005.05.003. 15939489

[ref2] Gada K.; Plant L. D. Two-pore domain potassium channels: emerging targets for novel analgesic drugs: IUPHAR Review 26. Br. J. Pharmacol. 2019, 176, 256–266. 10.1111/bph.14518. 30325008 PMC6295411

[ref3] Choe S. Potassium channel structures. Nat. Rev. Neurosci. 2002, 3, 115–121. 10.1038/nrn727. 11836519

[ref4] Zúñiga L.; Zúñiga R. Understanding the Cap Structure in K2P Channels. Front. Physiol. 2016, 7, 228 10.3389/fphys.2016.00228. 27378938 PMC4906011

[ref5] Doyle D. A.; Cabral J. M.; Pfuetzner R. A.; Kuo A.; Gulbis J. M.; Cohen S. L.; Chait B. T.; MacKinnon R. The Structure of the Potassium Channel: Molecular Basis of K^+^ Conduction and Selectivity. Science 1998, 280, 69 10.1126/science.280.5360.69. 9525859

[ref6] Heil B.; Ludwig J.; Lichtenberg-Fraté H.; Lengauer T. Computational recognition of potassium channel sequences. Bioinformatics 2006, 22, 1562–1568. 10.1093/bioinformatics/btl132. 16595554

[ref7] Goldstein S. A. N.; Bockenhauer D.; O’Kelly I.; Zilberberg N. Potassium leak channels and the KCNK family of two-p-domain subunits. Nat. Rev. Neurosci. 2001, 2, 175–184. 10.1038/35058574. 11256078

[ref8] Enyedi P.; Czirják G. Molecular Background of Leak K+ Currents: Two-Pore Domain Potassium Channels. Physiol. Rev. 2010, 90, 559–605. 10.1152/physrev.00029.2009. 20393194

[ref9] Aryal P.; Abd-Wahab F.; Bucci G.; Sansom M. S. P.; Tucker S. J. A hydrophobic barrier deep within the inner pore of the TWIK-1 K2P potassium channel. Nat. Commun. 2014, 5, 4377 10.1038/ncomms5377. 25001086 PMC4102122

[ref10] Dong Y. Y.; Pike A. C. W.; Mackenzie A.; McClenaghan C.; Aryal P.; Dong L.; Quigley A.; Grieben M.; Goubin S.; Mukhopadhyay S.; Ruda G. F.; Clausen M. V.; Cao L.; Brennan P. E.; Burgess-Brown N. A.; Sansom M. S. P.; Tucker S. J.; Carpenter E. P. K2P channel gating mechanisms revealed by structures of TREK-2 and a complex with Prozac. Science 2015, 347, 1256 10.1126/science.1261512. 25766236 PMC6034649

[ref11] Schewe M.; Nematian-Ardestani E.; Sun H.; Musinszki M.; Cordeiro S.; Bucci G.; de Groot; Bert L.; Tucker; Stephen J.; Rapedius M.; Baukrowitz T. A Non-canonical Voltage-Sensing Mechanism Controls Gating in K2P K^+^ Channels. Cell 2016, 164, 937–949. 10.1016/j.cell.2016.02.002. 26919430 PMC4771873

[ref12] Kopec W.; Rothberg B. S.; de Groot B. L. Molecular mechanism of a potassium channel gating through activation gate-selectivity filter coupling. Nat. Commun. 2019, 10, 5366 10.1038/s41467-019-13227-w. 31772184 PMC6879586

[ref13] Jorgensen C.; Darré L.; Oakes V.; Torella R.; Pryde D.; Domene C. Lateral Fenestrations in K^+^-Channels Explored Using Molecular Dynamics Simulations. Mol. Pharmaceutics 2016, 13 (7), 2263–2273. 10.1021/acs.molpharmaceut.5b00942. 27173896

[ref14] Oakes V.; Furini S; Pryde D.; Domene C. Exploring the Dynamics of the TWIK-1 Channel. Biophys. J. 2016, 111, 775–784. 10.1016/j.bpj.2016.07.009. 27558721 PMC5002071

[ref15] Roux B.; Bernèche S.; Egwolf B.; Lev B.; Noskov S. Y.; Rowley C. N.; Yu H. Ion selectivity in channels and transporters. J. Gen. Physiol. 2011, 137, 415–426. 10.1085/jgp.201010577. 21518830 PMC3082929

[ref16] Bernèche S.; Roux B. Molecular Dynamics of the KcsA K+ Channel in a Bilayer Membrane. Biophys. J. 2000, 78, 2900–2917. 10.1016/S0006-3495(00)76831-7. 10827971 PMC1300876

[ref17] Åqvist J.; Luzhkov V. Ion permeation mechanism of the potassium channel. Nature 2000, 404, 881–884. 10.1038/35009114. 10786795

[ref18] Luzhkov V. B.; Åqvist J. K+/Na+ selectivity of the KcsA potassium channel from microscopic free energy perturbation calculations. Biochim. Biophys. Acta, Protein Struct. Mol. Enzymol. 2001, 1548, 194–202. 10.1016/S0167-4838(01)00213-8. 11513964

[ref19] Bernèche S.; Roux B. Energetics of ion conduction through the K+ channel. Nature 2001, 414, 73–77. 10.1038/35102067. 11689945

[ref20] Bernèche S.; Roux B. A microscopic view of ion conduction through the K+ channel. Proc. Natl. Acad. Sci. U.S.A. 2003, 100, 8644 10.1073/pnas.1431750100. 12837936 PMC166365

[ref21] Domene C.; Sansom M. S. P. Potassium Channel, Ions, and Water: Simulation Studies Based on the High Resolution X-Ray Structure of KcsA. Biophys. J. 2003, 85, 2787–2800. 10.1016/S0006-3495(03)74702-X. 14581184 PMC1303560

[ref22] Holyoake J.; Domene C.; Bright J. N.; Sansom M. S. P. KcsA closed and open: modelling and simulation studies. Eur. Biophys. J. 2004, 33, 238–246. 10.1007/s00249-003-0355-2. 14574522

[ref23] Bernèche S.; Roux B. A Gate in the Selectivity Filter of Potassium Channels. Structure 2005, 13, 591–600. 10.1016/j.str.2004.12.019. 15837197

[ref24] Domene C.; Klein M. L.; Branduardi D.; Gervasio F. L.; Parrinello M. Conformational Changes and Gating at the Selectivity Filter of Potassium Channels. J. Am. Chem. Soc. 2008, 130, 9474–9480. 10.1021/ja801792g. 18588293

[ref25] Furini S.; Domene C. Atypical mechanism of conduction in potassium channels. Proc. Natl. Acad. Sci. U.S.A. 2009, 106, 16074 10.1073/pnas.0903226106. 19805261 PMC2752519

[ref26] Köpfer D. A.; Song C.; Gruene T.; Sheldrick G. M.; Zachariae U.; de Groot B. L. Ion permeation in K^+^ channels occurs by direct Coulomb knock-on. Science 2014, 346, 352 10.1126/science.1254840. 25324389

[ref27] Kutzner C.; Páll S.; Fechner M.; Esztermann A.; de Groot B. L.; Grubmüller H. More bang for your buck: Improved use of GPU nodes for GROMACS 2018. J. Comput. Chem. 2019, 40, 2418–2431. 10.1002/jcc.26011. 31260119

[ref28] Tribello G. A.; Bonomi M.; Branduardi D.; Camilloni C.; Bussi G. PLUMED 2: New feathers for an old bird. Comput. Phys. Commun. 2014, 185, 604–613. 10.1016/j.cpc.2013.09.018.

[ref29] Bonomi M.; Bussi G.; Camilloni C.; Tribello G. A.; Banáš P.; Barducci A.; Bernetti M.; Bolhuis P. G.; Bottaro S.; Branduardi D.; Capelli R.; Carloni P.; Ceriotti M.; Cesari A.; Chen H.; Chen W.; Colizzi F.; De S.; De La Pierre M.; Donadio D.; Drobot V.; Ensing B.; Ferguson A. L.; Filizola M.; Fraser J. S.; Fu H.; Gasparotto P.; Gervasio F. L.; Giberti F.; Gil-Ley A.; Giorgino T.; Heller G. T.; Hocky G. M.; Iannuzzi M.; Invernizzi M.; Jelfs K. E.; Jussupow A.; Kirilin E.; Laio A.; Limongelli V.; Lindorff-Larsen K.; Löhr T.; Marinelli F.; Martin-Samos L.; Masetti M.; Meyer R.; Michaelides A.; Molteni C.; Morishita T.; Nava M.; Paissoni C.; Papaleo E.; Parrinello M.; Pfaendtner J.; Piaggi P.; Piccini G.; Pietropaolo A.; Pietrucci F.; Pipolo S.; Provasi D.; Quigley D.; Raiteri P.; Raniolo S.; Rydzewski J.; Salvalaglio M.; Sosso G. C.; Spiwok V.; Šponer J.; Swenson D. W. H.; Tiwary P.; Valsson O.; Vendruscolo M.; Voth G. A.; White A. Promoting transparency and reproducibility in enhanced molecular simulations. Nat. Methods 2019, 16, 670–673. 10.1038/s41592-019-0506-8. 31363226

[ref30] Jensen M. Ø.; Borhani D. W.; Lindorff-Larsen K.; Maragakis P.; Jogini V.; Eastwood M. P.; Dror R. O.; Shaw D. E. Principles of conduction and hydrophobic gating in K^+^ channels. Proc. Natl. Acad. Sci. U.S.A. 2010, 107, 5833 10.1073/pnas.0911691107. 20231479 PMC2851896

[ref31] Pan A. C.; Cuello L. G.; Perozo E.; Roux B. Thermodynamic coupling between activation and inactivation gating in potassium channels revealed by free energy molecular dynamics simulations. J. Gen. Physiol. 2011, 138, 571–580. 10.1085/jgp.201110670. 22124115 PMC3226968

[ref32] Heer F. T.; Posson D. J.; Wojtas-Niziurski W.; Nimigean C. M.; Bernèche S. Mechanism of activation at the selectivity filter of the KcsA K(+) channel. eLife 2017, 6, e25844 10.7554/eLife.25844. 28994652 PMC5669632

[ref33] Georgoulia P. S.; Glykos N. M. Molecular simulation of peptides coming of age: Accurate prediction of folding, dynamics and structures. Arch. Biochem. Biophys. 2019, 664, 76–88. 10.1016/j.abb.2019.01.033. 30711540

[ref34] Furini S.; Domene C. Critical Assessment of Common Force Fields for Molecular Dynamics Simulations of Potassium Channels. J. Chem. Theory Comput. 2020, 16, 7148–7159. 10.1021/acs.jctc.0c00331. 33054190

[ref35] Fink M.; Lesage F.; Duprat F.; Heurteaux C.; Reyes R.; Fosset M.; Lazdunski M. A neuronal two P domain K+ channel stimulated by arachidonic acid and polyunsaturated fatty acids. EMBO J. 1998, 17, 3297–3308. 10.1093/emboj/17.12.3297. 9628867 PMC1170668

[ref36] Masetti M.; Berti C.; Ocello R.; Di Martino G. P.; Recanatini M.; Fiegna C.; Cavalli A. Multiscale Simulations of a Two-Pore Potassium Channel. J. Chem. Theory Comput. 2016, 12, 5681–5687. 10.1021/acs.jctc.6b00972. 27951666

[ref37] Brohawn S. G.; Campbell E. B.; MacKinnon R. Domain-swapped chain connectivity and gated membrane access in a Fab-mediated crystal of the human TRAAK K^+^ channel. Proc. Natl. Acad. Sci. U.S.A. 2013, 110, 2129 10.1073/pnas.1218950110. 23341632 PMC3568379

[ref38] Brohawn S. G.; Campbell E. B.; MacKinnon R. Physical mechanism for gating and mechanosensitivity of the human TRAAK K+ channel. Nature 2014, 516, 126–130. 10.1038/nature14013. 25471887 PMC4682367

[ref39] Brohawn S. G.; Su Z.; MacKinnon R. Mechanosensitivity is mediated directly by the lipid membrane in TRAAK and TREK1 K^+^ channels. Proc. Natl. Acad. Sci. U.S.A. 2014, 111, 3614 10.1073/pnas.1320768111. 24550493 PMC3948252

[ref40] Olsson M. H. M.; Søndergaard C. R.; Rostkowski M.; Jensen J. H. PROPKA3: Consistent Treatment of Internal and Surface Residues in Empirical pKa Predictions. J. Chem. Theory Comput. 2011, 7, 525–537. 10.1021/ct100578z. 26596171

[ref41] Jo S.; Kim T.; Iyer V. G.; Im W. CHARMM-GUI: A web-based graphical user interface for CHARMM. J. Comput. Chem. 2008, 29, 1859–1865. 10.1002/jcc.20945. 18351591

[ref42] Lee J.; Cheng X.; Swails J. M.; Yeom M. S.; Eastman P. K.; Lemkul J. A.; Wei S.; Buckner J.; Jeong J. C.; Qi Y.; Jo S.; Pande V. S.; Case D. A.; Brooks C. L.; MacKerell A. D.; Klauda J. B.; Im W. CHARMM-GUI Input Generator for NAMD, GROMACS, AMBER, OpenMM, and CHARMM/OpenMM Simulations Using the CHARMM36 Additive Force Field. J. Chem. Theory Comput. 2016, 12, 405–413. 10.1021/acs.jctc.5b00935. 26631602 PMC4712441

[ref43] Lomize M. A.; Pogozheva I. D.; Joo H.; Mosberg H. I.; Lomize A. L. OPM database and PPM web server: resources for positioning of proteins in membranes. Nucleic Acids Res. 2011, 40, D370–D376. 10.1093/nar/gkr703. 21890895 PMC3245162

[ref44] Jorgensen W. L.; Chandrasekhar J.; Madura J. D.; Impey R. W.; Klein M. L. Comparison of simple potential functions for simulating liquid water. J. Chem. Phys. 1983, 79, 926–935. 10.1063/1.445869.

[ref45] Huang J.; Rauscher S.; Nawrocki G.; Ran T.; Feig M.; de Groot B. L.; Grubmüller H.; MacKerell A. D. CHARMM36m: an improved force field for folded and intrinsically disordered proteins. Nat. Methods 2017, 14, 71–73. 10.1038/nmeth.4067. 27819658 PMC5199616

[ref46] Maier J. A.; Martinez C.; Kasavajhala K.; Wickstrom L.; Hauser K. E.; Simmerling C. ff14SB: Improving the Accuracy of Protein Side Chain and Backbone Parameters from ff99SB. J. Chem. Theory Comput. 2015, 11, 3696–3713. 10.1021/acs.jctc.5b00255. 26574453 PMC4821407

[ref47] Joung I. S.; Cheatham T. E. Determination of Alkali and Halide Monovalent Ion Parameters for Use in Explicitly Solvated Biomolecular Simulations. J. Phys. Chem. B 2008, 112, 9020–9041. 10.1021/jp8001614. 18593145 PMC2652252

[ref48] Essmann U.; Perera L.; Berkowitz M. L.; Darden T.; Lee H.; Pedersen L. G. A smooth particle mesh Ewald method. J. Chem. Phys. 1995, 103, 8577–8593. 10.1063/1.470117.

[ref49] Tuckerman M.; Berne B. J.; Martyna G. J. Reversible multiple time scale molecular dynamics. J. Chem. Phys. 1992, 97, 1990–2001. 10.1063/1.463137.

[ref50] Feller S. E.; Zhang Y.; Pastor R. W.; Brooks B. R. Constant pressure molecular dynamics simulation: The Langevin piston method. J. Chem. Phys. 1995, 103, 4613–4621. 10.1063/1.470648.

[ref51] Roux B. The Membrane Potential and its Representation by a Constant Electric Field in Computer Simulations. Biophys. J. 2008, 95, 4205–4216. 10.1529/biophysj.108.136499. 18641071 PMC2567939

[ref52] Gumbart J.; Khalili-Araghi F.; Sotomayor M.; Roux B. Constant electric field simulations of the membrane potential illustrated with simple systems. Biochim. Biophys. Acta, Biomembr. 2012, 1818, 294–302. 10.1016/j.bbamem.2011.09.030. PMC3575077 22001851

[ref53] Khalili-Araghi F.; Ziervogel B.; Gumbart J. C.; Roux B. Molecular dynamics simulations of membrane proteins under asymmetric ionic concentrations. J. Gen. Physiol. 2013, 142, 465–475. 10.1085/jgp.201311014. 24081985 PMC3787774

[ref54] Phillips J. C.; Braun R.; Wang W.; Gumbart J.; Tajkhorshid E.; Villa E.; Chipot C.; Skeel R. D.; Kalé L.; Schulten K. Scalable molecular dynamics with NAMD. J. Comput. Chem. 2005, 26, 1781–1802. 10.1002/jcc.20289. 16222654 PMC2486339

[ref55] Michaud-Agrawal N.; Denning E. J.; Woolf T. B.; Beckstein O. MDAnalysis: A toolkit for the analysis of molecular dynamics simulations. J. Comput. Chem. 2011, 32, 2319–2327. 10.1002/jcc.21787. 21500218 PMC3144279

[ref56] Gowers R.; Linke M.; Barnoud J.; Reddy T.; Melo M.; Seyler S.; Domański J.; Dotson D.; Buchoux S.; Kenney I.; Beckstein O. MDAnalysis: A Python Package for the Rapid Analysis of Molecular Dynamics Simulations; Los Alamos National Lab: Los Alamos, NM, 2016; pp 98–105.

[ref57] Wickham H. ggplot2: Elegant Graphics for Data Analysis; Springer Publishing Company, 2009.

[ref58] R: A Language and Environment for Statistical Computing; R Core Team: Vienna, 2020.

[ref59] Ceriotti M.; Tribello G. A.; Parrinello M. Simplifying the representation of complex free-energy landscapes using sketch-map. Proc. Natl. Acad. Sci. U.S.A. 2011, 108, 13023 10.1073/pnas.1108486108. 21730167 PMC3156203

[ref60] Ceriotti M.; Tribello G. A.; Parrinello M. Demonstrating the Transferability and the Descriptive Power of Sketch-Map. J. Chem. Theory Comput. 2013, 9, 1521–1532. 10.1021/ct3010563. 26587614

[ref61] Mu Y.; Nguyen P. H.; Stock G. Energy landscape of a small peptide revealed by dihedral angle principal component analysis. Proteins 2005, 58, 45–52. 10.1002/prot.20310. 15521057

[ref62] Altis A.; Nguyen P. H.; Hegger R.; Stock G. Dihedral angle principal component analysis of molecular dynamics simulations. J. Chem. Phys. 2007, 126, 244111 10.1063/1.2746330. 17614541

[ref63] Lolicato M.; Riegelhaupt; Paul M.; Arrigoni C.; Clark; Kimberly A.; Minor; Daniel L. Jr. Transmembrane Helix Straightening and Buckling Underlies Activation of Mechanosensitive and Thermosensitive K_2P_ Channels. Neuron 2014, 84, 1198–1212. 10.1016/j.neuron.2014.11.017. 25500157 PMC4270892

[ref64] McClenaghan C.; Schewe M.; Aryal P.; Carpenter E. P.; Baukrowitz T.; Tucker S. J. Polymodal activation of the TREK-2 K2P channel produces structurally distinct open states. J. Gen. Physiol. 2016, 147, 497–505. 10.1085/jgp.201611601. 27241700 PMC4886281

[ref65] Lolicato M.; Arrigoni C.; Mori T.; Sekioka Y.; Bryant C.; Clark K. A.; Minor D. L. K2P2.1 (TREK-1)–activator complexes reveal a cryptic selectivity filter binding site. Nature 2017, 547, 364–368. 10.1038/nature22988. 28693035 PMC5778891

[ref66] Douguet D.; Honoré E. Mammalian Mechanoelectrical Transduction: Structure and Function of Force-Gated Ion Channels. Cell 2019, 179, 340–354. 10.1016/j.cell.2019.08.049. 31585078

[ref67] Harrigan M. P.; McKiernan K. A.; Shanmugasundaram V.; Denny R. A.; Pande V. S. Markov modeling reveals novel intracellular modulation of the human TREK-2 selectivity filter. Sci. Rep. 2017, 7, 632 10.1038/s41598-017-00256-y. 28377596 PMC5428000

[ref68] Brennecke J. T.; de Groot B. L. Mechanism of Mechanosensitive Gating of the TREK-2 Potassium Channel. Biophys. J. 2018, 114, 1336–1343. 10.1016/j.bpj.2018.01.030. 29590591 PMC5883942

[ref69] Öster C.; Hendriks K.; Kopec W.; Chevelkov V.; Shi C.; Michl D.; Lange S.; Sun H.; de Groot B. L.; Lange A. The conduction pathway of potassium channels is water free under physiological conditions. Sci. Adv. 2019, 5, eaaw6756 10.1126/sciadv.aaw6756. 31392272 PMC6669007

[ref70] Blin S.; Ben Soussia I.; Kim E.-J.; Brau F.; Kang D.; Lesage F.; Bichet D. Mixing and matching TREK/TRAAK subunits generate heterodimeric K_2P_ channels with unique properties. Proc. Natl. Acad. Sci. U.S.A. 2016, 113, 4200 10.1073/pnas.1522748113. 27035965 PMC4839434

