# Supplementary material for: Conduction and Gating Properties of the TRAAK Channel from Molecular Dynamics Simulations with Different Force Fields
Source: J Chem Inf Model. 2020 Dec 9;60(12):6532–43. doi: 10.1021/acs.jcim.0c01179 (PMC8016162; doi:10.1021/acs.jcim.0c01179)
Supplement: Supplementary file 1 — ci0c01179_si_001.pdf [file ci0c01179_si_001.pdf]

SUPPORTING INFORMATION

Conduction and Gating Properties of the TRAAK Channel from Molecular Dynamics Simulations with Different Force Fields

Riccardo Ocello,[1] Simone Furini,[2] Francesca Lugli,[3] Maurizio Recanatini,[1] Carmen Domene[4,5] and Matteo Masetti[1]**

[1]Department of Pharmacy and Biotechnology, Alma Mater Studiorum − Università di Bologna, via Belmeloro 6, 40126 Bologna, Italy. [2]Department of Medical Biotechnologies, University of Siena, 53100 Siena, Italy. [3]Department of Chemistry "G. Ciamician", Alma Mater Studiorum − Università di Bologna, via Selmi 2, 40126 Bologna, Italy. [4]Department of Chemistry, University of Bath, Claverton Down, Bath, BA2 7AY, UK. [5]Department of Chemistry, University of Oxford, Mansfield Road, Oxford, OX1 3TA, UK.

Table S1. Relative population of the main clusters identified from the projection of each subset of trajectories onto the Sketch-Map low-d space considering either the KK or the KWK ion traslocation mechanisms and using the AMBER or CHARMM force fields.

AMBER-KK		AMBER-KWK		CHARMM-KK		CHARMM-KWK	
**Cluster #**	**Population (%)**	**Cluster #**	**Population (%)**	**Cluster #**	**Population (%)**	**Cluster #**	**Population (%)**
1	97.8	1	43.2	1	52.3	1	35.5
		2	16.6	2	10.3	2	11.6
		3	13.3	3	7.8	3	7.4
		4	11.0	4	7.6	4	6.9
		5	8.5	5	7.1	5	5.3
		6	4.6	6	4.0	6	4.8
		7	0.9	7	0.7	7	4.6
				8	0.3	8	3.5
				9	0.2	9	2.7
						10	0.7
**TOTAL:**	97.8		98.1		90.3		83.0

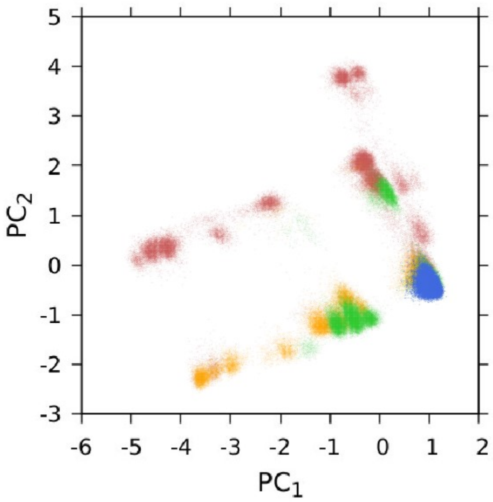

Figure S1. Principal Component Analysis carried out on the degrees of freedom of the selectivity filter. Blue, green, orange, and red correspond to data from AMBER-KK, AMBER-KWK, CHARMM-KK, and CHARMM-KWK simulations respectively.

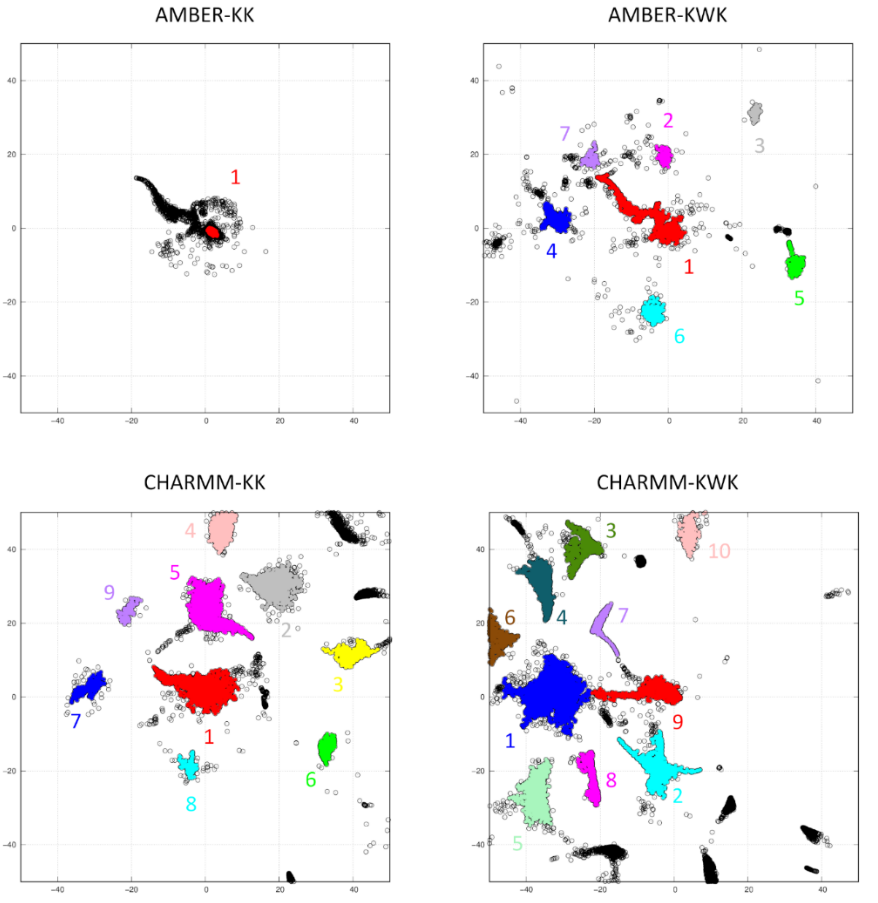

Figure S2. Density-based cluster analysis performed on the projection of the trajectories of each subset of simulations onto the low-d space identified by Sketch-Map. The most populated clusters are color-coded and numbered according to information presented in Table S1.

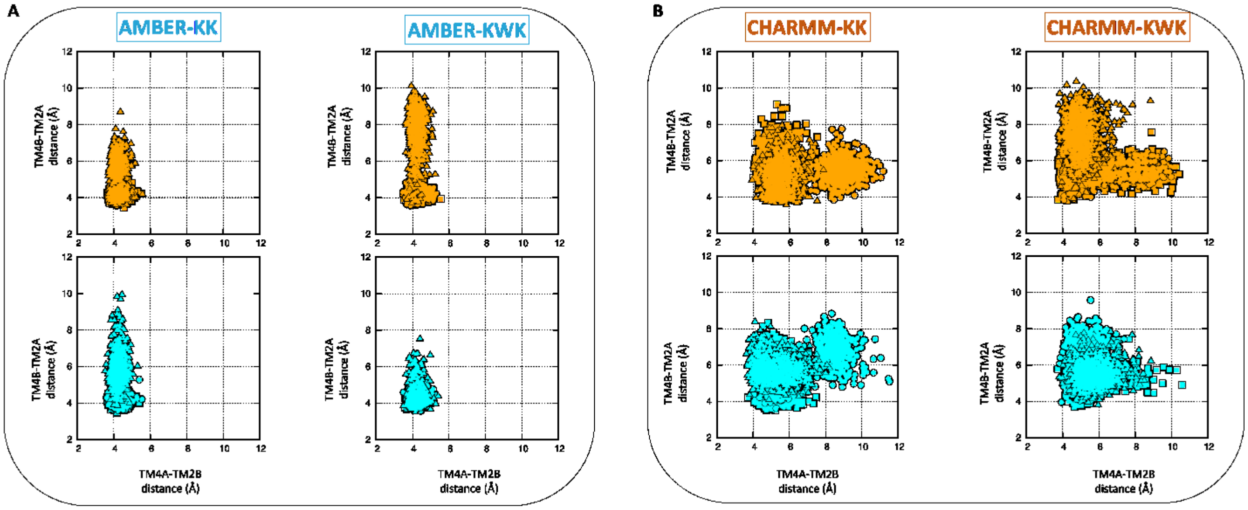

Figure S3. Scatter plot of the TM4-TM2 distances where cyan and orange dots correspond to the simulation sets at +100 and +200 mV, respectively, with the AMBER (panel A) and CHARMM (panel B) force fields. Squares, circles and triangles correspond to run #1, 2, and 3, respectively.

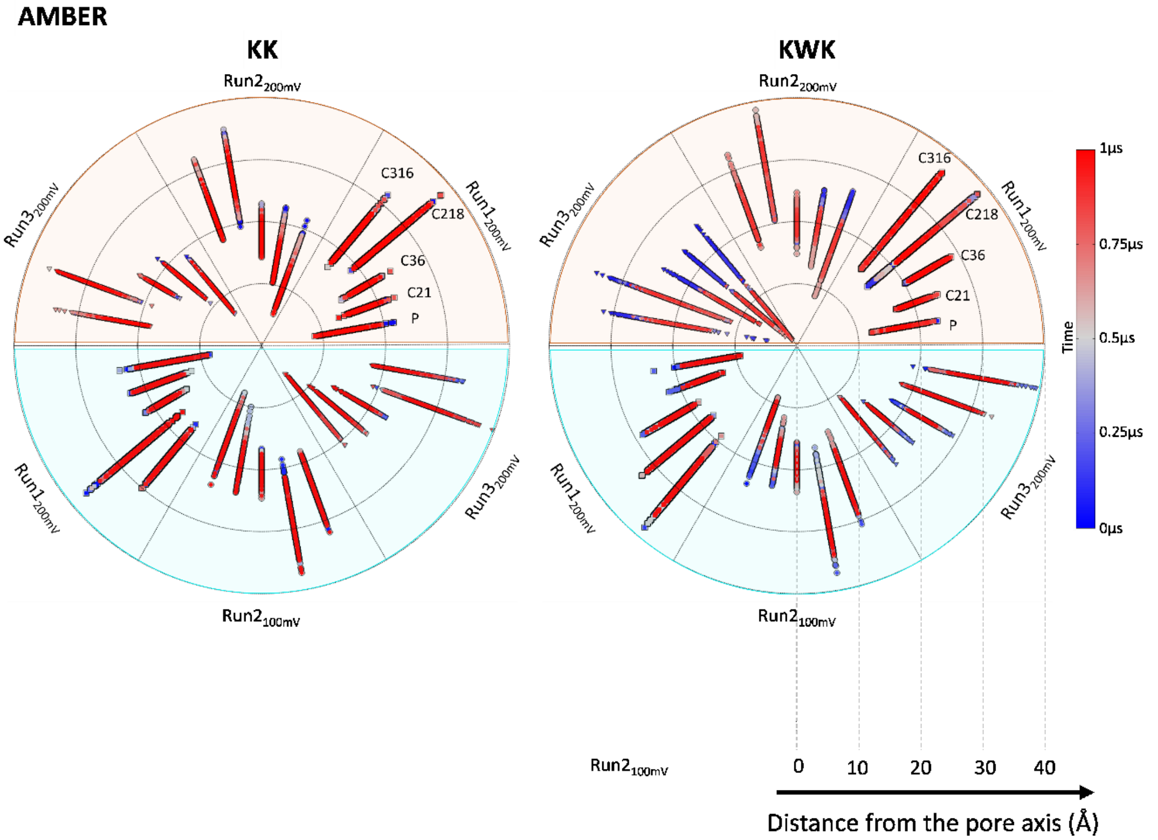

Figure S4. Time evolution of lipids entering the cavity with the AMBER force field. The five datasets for each run corrspond to the distances sampled by the lipid atoms shown in Figure 5b of the main manuscript and are colored according to the simulation time as shown in the colorbar on the right.

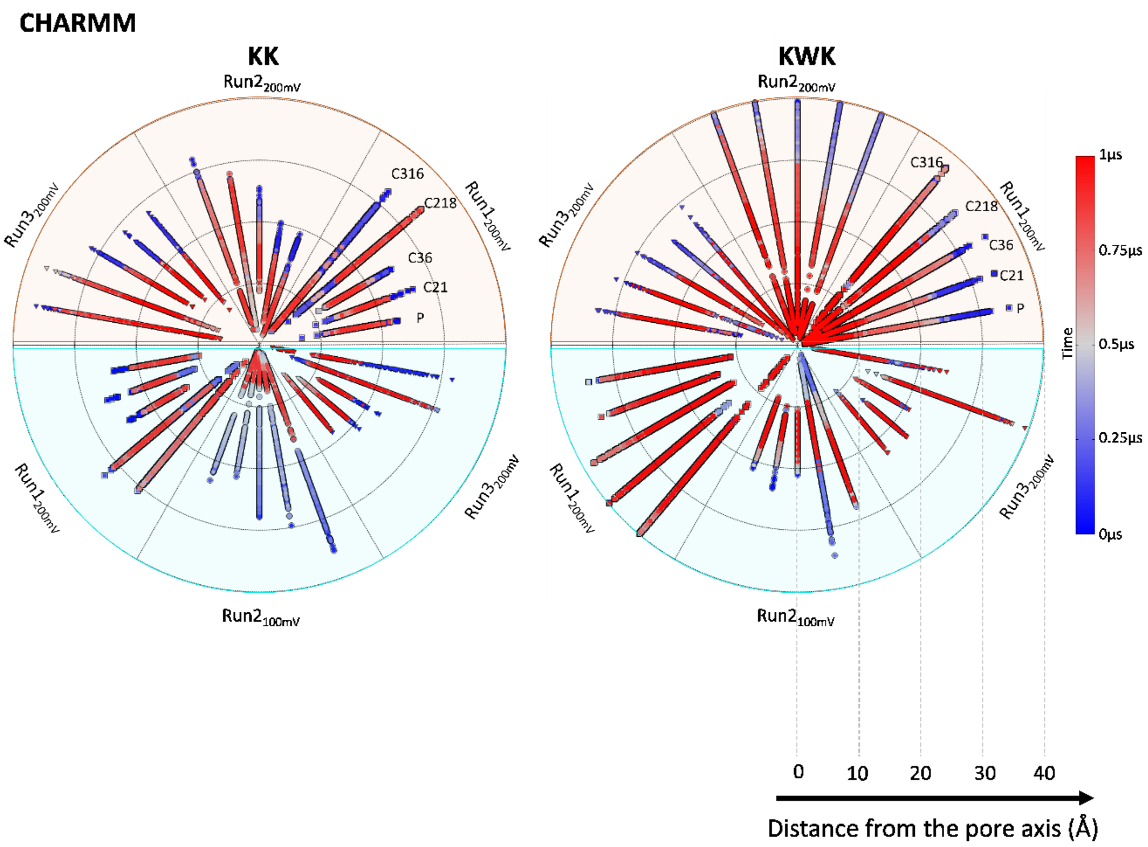

Figure S5. Time evolution of lipids entering the cavity with the CHARMM force field. The five datasets for each run corrspond to the distances sampled by the lipid atoms shown in Figure 5b of the main manuscript and are colored according to the simulation time as shown in the colorbar on the right.
